# Diet-Regulated Anxiety

**DOI:** 10.1155/2013/701967

**Published:** 2013-08-20

**Authors:** Michelle Murphy, Julian G. Mercer

**Affiliations:** Rowett Institute of Nutrition and Health, University of Aberdeen, Greenburn Road, Bucksburn, Aberdeen AB21 9SB, UK

## Abstract

A substantial proportion of noncommunicable disease originates in habitual overconsumption of calories, which can lead to weight gain and obesity and attendant comorbidities. At the other end of the spectrum, the consequences of undernutrition in early life and at different stages of adult life can also have major impact on wellbeing and quality of life. To help address some of these issues, greater understanding is required of interactions with food and contemporary diets throughout the life course and at a number of different levels: physiological, metabolic, psychological, and emotional. Here we review the current literature on the effects of dietary manipulation on anxiety-like behaviour. This evidence, assembled from study of preclinical models of diet challenge from gestation to adult life, supports a role for diet in the important connections between psychology, physiology, and behaviour. Analogous processes in the human population in our current obesogenic environment are likely to contribute to individual and societal challenges in this area.

## 1. Introduction 

The quality and composition of the food we eat is under constant scrutiny, and there are numerous factors which affect our food selection and preferences. The disparate properties of the food we consume themselves act in a regulatory feedback system to impact on our future choices. One way in which food may influence subsequent choice is by affecting mood and consequently behaviour. Although it is recognised that mood can affect what we eat, here we consider how food affects our mood and, in particular, anxiety levels. 

The fat and sugar content of contemporary diets and of processed foods, in particular, is a high profile issue for consumer groups, the media and health professionals. There is also increasing pressure from public health bodies and governments to reduce the amounts of fats and sugars, along with salt, in our diet. However, there may be unexpected consequences of such changes as our bodies may respond not only to the presence of these substances in our diets but also their absence, following a dietary manipulation. Withdrawal of dietary components has the potential to influence future food choices and also our emotional health. Generic, calorie restriction, and weight loss diets usually fail to produce the desired long-term outcomes, but increased awareness of the effects of the composition of our diet over both the short and the long term may increase our ability to adapt our diets successfully to improve both metabolic and emotional wellbeing.

Anxiety is linked to prevalence of disease [1]. People with mood disorders often have poor quality diets which are low in fruits and vegetables but high in fat and sugar [2]. Increasingly, modified diets are being used to treat behavioural and mood disorders such as attention deficit disorder, where diets low in sugar and high in fatty acids are recommended [3]. Children have less control over their food choice than adults as they are highly influenced by their parents [4]. However, children do have a reputation for knowing what they want to eat and being “picky” eaters, and high levels of anxiety have been shown to increase selective or fussy behaviour [5]. Dietary choice, whether it is quality/composition or quantity, is also affected in overweight adults who report increased calorie intake when they are under stress [6]. Stress can interact with eating behaviour in a number of ways regardless of an individual's normal eating habits [7]. 

Nutrition and anxiety can have both independent and interactive negative impacts on health. The life stage at which either of these factors becomes unbalanced impacts on the severity of the outcome. Consequently, insults which occur during critical sensitive stages in development, especially *in utero* or in early life may have long-lasting consequences. This emphasises the importance of maternal diet and diet during adult life, and their interaction with mental health. The aim of this review is to bring together existing knowledge of how food components affect anxiety at various stages of development, while highlighting some major gaps in our current understanding.

## 2. Measures of Anxiety

Tests for anxiety focus mainly on an animal's normal response to a novel environment or object(s) and measure changes in locomotion, exploration, and reactive behaviour (e.g., preference for enclosed and dark environments) as well as changes in acute behaviour such as grooming and rearing (see Table 1 for common tests). Tests for anxiety tend to be less challenging to the animal than those for depression, which often attempt to enforce behaviour such as swimming or an escape response. Detailed information on behavioural tests for anxiety and the differences between anxiety and depression tests can be found elsewhere [8]. A wide variety of behavioural tests are used in drug testing but, in studies investigating dietary effects, elevated plus maze (EPM) and open field (OF) are the tests most frequently deployed. By their very nature these tests are affected by subtle variations in setting, manipulation, and indeed the strain or sex of the animal. Detailed evaluations of the more common maze tests are available [9, 10], setting out the limitations of the tests and differences between them. It is difficult to compare results between tests since exposure time, habituation, and prior experience of the test are all likely to affect the outcomes. 

## 3. Effects of Diet

Concern over obesity and metabolic disease, as well as their potential relationships with mental health disorders, has led to increasing emphasis on strategies to tackle poor eating habits. This has led researchers to investigate the emotional value of food, which may make an important contribution to diet choice [11, 12]. Dietary habits at various stages of life from gestation into adulthood have been connected not only to disruption of energy balance but also to attention, mood, behavior, and anxiety disorders. 

### 3.1. Maternal Nutrition

Increased risk of developing numerous conditions including obesity, metabolic disease, mental health issues, and anxiety has been linked to poor early nutrition, particularly during critical stages of *in utero* development [13]. One example of a mental disorder which has been linked to obesity is attention deficit/hyperactivity disorder (ADHD), where mothers of children with the condition are twice as likely to be obese as mothers of children without the condition [14, 15], illustrating that maternal nutrition may affect the mental health and behaviour of offspring. However, there are likely to be confounding issues related to the mother's health in these studies, and this supports preclinical work currently being undertaken to identify the association between mental health and maternal nutrition as in animal models health can be closely monitored and food intake and diet closely regulated.

Various maternal diets in rodents have been shown to impact on circulating hormone levels which are likely to contribute to increased susceptibility to disease. Maternal high-fat diets increased circulating leptin and insulin levels [16], as well as desensitising the dopamine system [17], which affected the offspring's feeding behaviour. Another study using maternal high-fat (saturated or trans fat) feeding from 4 weeks prior to mating through until weaning, showed that while a diet high in saturated fat increased circulating leptin levels in both male and female offspring, anxiety, as assessed on the elevated plus maze (EPM), was increased only in male rats and apparently irrespective of the composition of maternal high-fat feeding [18]. This suggests that behavioural effects may have some sex specificity although this requires further investigation. Links between maternal diet and anxiety have also been associated with the mood regulating neurotransmitter, serotonin [19]. Peleg-Raibstein et al. showed increased anxiety in the EPM in a model of maternal high fat-feeding (dams fed 60% high-fat diet prior to mating through to weaning) and also suggest that this effect on anxiety is due to elevated serotonin in the ventral hippocampus as a result of the maternal high-fat feeding [20]. Again using 60% high-fat diet prior to mating through until weaning, Sasaki et al. showed that males decreased time in centre of OF, females spent less time in open arms of EPM, but no differences were seen in the light dark box [21]. In terms of hormonal changes, this study also reported that offspring from dams on a high-fat diet had reduced corticosterone levels while the corticosterone receptors had increased abundance in the amygdala of females only [21]. 

**Figure 1:**
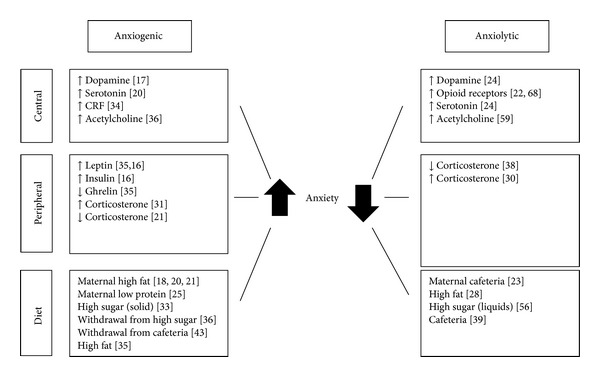
Factors implicated in mediating the effects of diet on anxiety. This illustrates some of the conflicting results from various palatable diet regimes and shows the complexity in deducing the pathways by which diets regulate anxiety levels. Numbers refer to associated referenced work.

Maternal diets composed of mixed palatable “junk” food or cafeteria feeding have been demonstrated to increase expression of opioid receptors and reduce dopamine transporters in offspring during early life. Although this effect appears to be reversible in later life, it suggests that the maternal diet is responsible for reduced sensitivity of opioid and dopamine signalling [22]. Also focusing on the effects of cafeteria diet, Wright et al. showed that dams fed cafeteria diet from weaning to mating produced male offspring that had reduced anxiety, increasing open arm exploration in the EPM and reducing latency to enter the centre of the open field [23]. Even providing this diet during the lactation phase only influenced dopamine and serotonin levels of the offspring [24]. There are numerous reasons why a cafeteria style diet might have different consequences to the high-fat diets described above. For instance, Figure 1 shows some of the central and peripheral factors which are affected by maternal diet and have been implicated as potential mediators of diet-regulated anxiety. The potentially high-sugar content of a mixed cafeteria style diet may also be responsible for counteracting the effects of the fat content or perhaps the more defining characteristic of a cafeteria diet; namely, the high variability and increased novelty and choice of food types may allow individuals to adapt their diet consciously or subconsciously to best suit their requirements to minimise anxiety.

Perturbations in supply of other macronutrients during early life may also impact on emotional behaviour, with low consumption of key dietary components such as protein affecting anxiety behaviour of offspring. For example, one study showed that protein restriction during pregnancy, but not lactation, increased anxiety as measured by EPM and decreased motivation for sucrose solution in operant system, but without effect on sucrose preference when given free access [25]. Likewise, another study of protein restriction from lactation onwards using foster dams found that anxiolytic drugs had little differential effect on anxiety of these animals in the EPM compared to rats on a standard diet, suggesting that any effects of malnutrition, in particular protein restriction, are likely to occur earlier in development [26]. 

### 3.2. High Fat

Alsiö et al. showed an inverse relationship between high-fat feeding of rats and anxiety in the EPM [27], and this was supported by A. Prasad and C. Prasad who found that diets high in fat but not those high in carbohydrates or protein were anxiolytic after just 1 week of dietary intervention [28]. In the light dark box paradigm, mice on a high-fat diet showed no response to an enforced stress whereas mice on a low-fat diet reduced the time spent in the light box by approximately 30% [29]. 

By contrast, Buchenauer et al. describe a reduced level of activity with changes in generic behaviour such as rearing (decreased) and grooming (increased) as indicators of increased anxiety in rats following exposure to a 35% high-fat diet for 8 weeks [30]. Likewise, mice fed a 58% high-fat diet for 12 weeks in order to promote obesity have shown increased corticosterone prior to and following a stressor as well as anxiogenic responses in OF and EPM [31]. This raises the issue of the implications of dietary regime on baseline anxiety and on responses to subsequent exposure to stressful experiences. 

As is frequently the case with dietary manipulations, the precise composition of the diet may be an important variable influencing the experimental outcomes. Accordingly, it has been suggested that saturated fatty acids are less anxiogenic than trans fats [32], so the source of fat in the diet may be as important as the quantity given. As indicated briefly above, both the quantity and type of fat in the diet vary across current reports on anxiety, along with the duration of feeding, all of which may impact on the anxiolytic or anxiogenic outcomes of high-fat diet feeding. 

### 3.3. High Sugar

Solid chow diet enriched with sucrose appears to increase anxiety in rats as indicated by decreased time spent in the light chamber of a light-dark box although there was no effect on OF exploration [33]. 

Little work has been done on the direct anxiogenic properties of sugar in the diet although there is more information on the effects of subsequent withdrawal of sugar. Withdrawal of a liquid high-sugar chocolate-flavoured diet in rats increased anxiety on the EPM and also increased corticotropin-releasing factor (CRF) expression in the amygdala [34]. Interestingly, these rats were also hypophagic on standard chow. This same diet, even if provided for a short period of time (10 minutes) following a period of food deprivation, induced changes in hormonal levels and behaviour, with increased leptin, decreased ghrelin, and increased anxiety as measured in the EPM [35] suggesting that the anxiogenic effects of a palatable diet are fast acting and seen almost immediately after presentation of the food. The work of Avena and Hoebel and colleagues on withdrawal from sugar has been widely cited as a model of food addiction. With this model, rats habituated to intermittent feeding on sucrose were less willing to enter the open arms of the EPM after sucrose had been withheld suggesting an increased state of anxiety similar to that seen during withdrawal from addictive drugs [36]. It is interesting to note that this work has been done on withdrawal from sweetened liquids, and the form and schedule in which sugar is presented may affect quantities consumed and the extent of effects on anxiety. 

Longer-term effects of a high-sugar diet were described by Souza et al. [33] who reported that after 4 months rats showed increased anxiety in the light/dark box but no effects in the OF setting. This again raises the question of the comparability of results from different behavioural paradigms and whether experimental examination of a greater range of tests could be revealing.

### 3.4. Mixed Palatable Diets

High-energy diets with a mixed composition of fat and sugar are known to result in memory deficits [37]. However, due to what may be opposing effects of fat and sugar on anxiety, as outlined above, it is difficult to predict their impact on behaviour and anxiety, and limited work has been done in this area.

Exposure to a “comfort” diet enriched with sugar and fat for just 6 days decreased corticosterone levels in rats following a foot shock stressor but did not affect basal levels. Anxiety behaviour was also decreased in an OF arena but no effects were observed either before or after stress in the EPM [38]. Likewise, stress responses to neonatal handling and early life isolation can be ameliorated by the anxiolytic effects of palatable diet high in fat and sugar [39], with increased locomotion and reduced anxiety in both the EPM and OF paradigms. This was confirmed in other work where stress was induced by isolation (maternal separation) and high-fat diets were reported to ameliorate anxiety in the EPM and light dark box in both dams [41] and pups [40–42]. It may be important that the diets were given in a cafeteria style and were also high in sugar. 

Withdrawal of palatable (high fat, high sugar) diets has been shown to increase anxiety in obesity-prone animals in the OF paradigm as well as to increase motivation (operant lever pressing) for sucrose pellets compared to animals on the same feeding schedule but classed as being obesity resistant based on relative weight gain [43]. This would support the theory that preference for a diet or predisposition to consume a higher intake may interact with the primary diet effect and that effects on anxiety might be influenced by body phenotype rather than being solely diet dependent. Essentially, it appears that anxiety and diet and energy balance are likely to be interrelated. This will complicate attempts to distinguish between cause and effect, particularly with a mixed cafeteria style diet. 

### 3.5. Irregular Eating

It is not only the components of the diet that affect anxiety behaviour. The frequency and regularity of feeding also affect circulating hormones and impact on behaviour due to effects on the circadian rhythms of the regulatory feeding and reward systems [44]. Models of irregular feeding can be considered to mimic the sporadic eating habits of dieters. Feeding a mixed high fat, high sugar diet to rats on an intermittent schedule either 2 hours a day or 3 days a week blocked the elevation of corticosterone associated with restraint stress seen in rats fed the palatable diet ad lib [45]. Rats given alternating access to a preferred high-sugar chocolate-flavoured diet had decreased activity and decreased time in the open arm of the EPM upon withdrawal from the preferred high sugar diet [46], and anxiety behaviour was greatest in those animals with the greatest propensity to binge. This may reinforce a cycle where mood or anxiety determines intake and the availability of certain foods feeds back on mood and behaviour.

## 4. Mechanisms 

There are a number of possible mediators of the effect of diet on anxiety-like behaviour, including both peripheral and central mediators such as corticosteroids, insulin leptin, acetylcholine, serotonin, opioids and dopamine. The potential contribution of these factors is considered here in relation to the various palatable diets discussed previously. 

The ability of maternal environment to influence the health status of offspring has been linked to various physiological changes associated with both over- and undernutrition of the mother [47]. There is evidence that hormones associated with energy status of the mother such as insulin and leptin as well as expression of neurotransmitters associated with reward such as dopamine can impact on anxiety and other behaviour disorders of offspring [48]. Also, the disregulation of serotonin, which also plays an important role in regulating neurogenesis and synaptogenesis and is affected by diet and stress [49], has been strongly linked to effects of maternal high-fat diet on anxiety in offspring [19, 50]. It has also been suggested that undernutrition of the mother alters the HPA axis of the offspring [51] and increases corticosterone [52], and these changes have all been associated with mediating the anxiety phenotype of the offspring. All of these factors may contribute to the overall impact that maternal diet can have on the anxiety levels of offspring and associated behaviour. Nutritional insult in early life may also influence the developing brain and behaviour and mental health in later life via epigenetic programming mechanisms [53]. 

In adults there are a number of common components of the stress, reward, and homeostatic pathways that link feeding behaviour, dietary choice, and anxious behaviour. While the hypothalamus is classically known for its role in the regulation of food intake and energy balance, corticosteroid receptors in this part of the brain are also known to be involved in the stress response, and this may provide a link between mood or anxiety levels and macronutrient preference or intake quantities [54, 55]. Circulating corticosterone is commonly measured as an indicator of stress and has been reported several times to be influenced by diet. Although it may be difficult to distinguish whether this is a result of rapid weight gain or a particular dietary component, the speed of the response would suggest the latter. A study by Ortolani et al. showed attenuation of corticosterone induction following stress after just 6-day exposure to a palatable diet compos of high fat and high sugar cafeteria style food although no effects of diet were seen on basal corticosterone levels [38]. Similarly, exposure to 30% sucrose solution had no effect on basal corticosterone but suppressed the anxiogenic effects of restraint stress [56]. By contrast, Buchenauer et al. describe elevated corticosterone levels in diet-induced obese rats following 8 weeks on a high fat-diet [30]. In mice there were no effects of HF diet on the HPA axis following a forced swim stress [29] although the diet did protect against stress-(social defeat) induced anxiety and depressive behaviour. Overall, this body of data appears to suggest that diet itself does not generically affect corticosterone or stress levels but may mediate the (protective) response to additional anxiogenic environments or stresses. 

High-fat diets result in alterations to the circulating levels of insulin, and the resulting resistance to leptin has been associated with cognitive deficits in mice [57]. In a similar manner, mice provided with a 10% sucrose solution also became insulin resistant and had impaired cognitive function [58].

Morganstern et al. suggest a role for acetylcholine in the mediation of the effect of high-fat diet in reducing anxiety. These studies showed that high-fat diet increased open arm entries in the EPM and increased exploration of a novel object but, interestingly, also induced a reduction in acetylcholine esterase suggesting that high fat diet may increase neurotransmission via acetylcholine in the frontal cortex and hypothalamus [59]. In terms of sugar intake, acetylcholine has also been implicated in the generation of anxiety. Avena et al. described elevated acetylcholine levels in the nucleus accumbens as well as suppressed levels of dopamine, and suggested that these may be responsible for increased anxiety following withdrawal from sucrose [36]. Review of various types of anxiety disorders in humans and rodents has identified similarities in the activation of neuronal pathways in the medial prefrontal cortex [60, 61]. 

The central feeding pathways are known to contain orexigenic and anorexigenic neuropeptides which are responsive to changes in diet and energy status, but their role in behaviour, mood, and, indeed, anxiety regulation is less well understood. Recently, it has been shown that stress which results in anxious behaviour increases activation of POMC and AGRP in arcuate neurons [62], while ablation of POMC neurons in the hypothalamus creates a phenotype with increased anxiety-related behaviour on the EPM [63]. There is also some evidence that treatment with NPY can influence anxiety although contradictory findings make it difficult to determine the role it may play [64]. This would suggest that there is some potential for “traditional” feeding pathways to also mediate anxiety-related behaviour in response to diet.

Opioid pathways are strongly involved in preference for certain types of food, and, in a study where rats were divided into groups based on fat or sugar preference, responses differed depending on the opioid system being challenged [65]. Injection of opioid agonists increased fat preference, but when sweet foods were preferred over high-fat foods, neither the agonist, DAMGO, nor the antagonist, naltrexone, injected into the PVN affected the amount of preferred food consumed [65]. However, in a similar study looking at rats that had developed a food preference, the antagonist, naltrexone, injected into the amygdala, suppressed intake of the preferred food without affecting intake from other foods [66]. *μ*-opioid receptors have also been shown to play a crucial role in motivation for and liking of sugar (sucrose) and fat (corn oil), where an antagonist injected into the nucleus accumbens shell of rats produced fully reversible changes in feeding behaviour [67]. Likewise, opioids have a known role in anxiety; activation of delta opioid receptors suppresses anxious behaviour in the EPM [68]. For a review on the role of opioids in anxiety, see [69]. This confirms a dual role of opioids in mediating the intake of palatable foods and in regulating anxiety, making them likely facilitators of the effects of high-fat and high-sugar foods on anxiety-related behaviour.

Levels of dopamine receptor (D) expression within the nucleus accumbens are responsive to high-fat diet, with D1 levels decreasing and D2 levels increasing [31]. This would suggest that the ratio of these receptors may be important in mediating what is, in this case, an anxiogenic effect of high-fat diet. High-sugar diets have also been found to affect dopamine receptor expression. In a model of intermittent exposure to sucrose Spangler et al. showed that there was also variation in the response of dopamine receptors in the nucleus accumbens to high-sugar consumption which decreased D2 and increased levels of D3 [70].

Figure 1 shows a summary of some of the potential mediators involved in diet-anxiety interactions. This highlights that there appear to be interactions between classical homeostatic feeding pathways, both peripheral and central, stress pathways, and central reward pathways in response to both diet and anxiety, which then give rise to behavioural outcomes. The exact relationships are however still unclear and further research is needed to identify the specific involvement of different brain regions and the hormones and neurotransmitters involved in these interactions. 

## 5. Discussion

The effects of mood on eating habits and preferences have been investigated at a number of levels and over an extended period [71], but it is only recently that the focus of such investigation has been turned on its head through examination of the effects of diet and food components on mood, behavior, and future dietary preferences. Here, we have examined the effects of diet on anxiety-related behaviour and have highlighted that foods high in fat and/or sugar, or that are highly palatable, have the potential to impact on behaviour in animal models and, by extrapolation, in humans. Whether food modulates anxiety through anxiolytic or anxiogenic actions is likely to depend not only on its composition, but also on previous dietary experiences and maternal diet during and prior to pregnancy as well as contemporary stress-inducing experiences. This makes the role of food in regulating anxiety a highly complex topic, but also an area where increased understanding could lead to the development of strategies to use and adapt food and eating behaviour to beneficially address metabolic and mood disorders. 

Currently, it appears that high fat is anxiolytic while diets high in sugar may have more anxiogenic characteristics. In terms of human lifestyle choices the situation is likely to be less clear cut, and mixed palatable diets are perhaps more relevant. Typically therefore, it is these diets which offer the greatest challenge of interpretation and manipulation, being highly variable in composition. Nevertheless, there is potential for these diets to be optimised and designed with beneficial effects for anxiety and general health.

Variation in mood affects food choice, shifting food preference towards foods high in fat and sugar [72], but, in the light of the evidence discussed above, this modification in preference may lead in turn to changes in anxiety and mood, thereby creating a feedback cycle. It is possible that in the wild, and across human evolution, consuming a high energy diet and being “cautious” could constitute beneficial behaviour conferring survival advantage and ensuring ingestion of sufficient energy supplies while reducing risk of predation. It can be speculated that ingestion of an anxiogenic food could lead to increased wariness and ingestion of familiar foods, whereas anxiolytic foods could increase exploration of the environment, and discovery of new palatable foods. Either scenario could have the potential to change behaviour and the mix of macronutrients and energy in the diet. In modern society with its wide range of food choice but where obesity and anxiety disorders are increasing, increased understanding of relationships with food at psychological and physiological levels including the associations between dietary choice and mood may be relevant to longer-term metabolic and mental health and be amenable to manipulation. 

The research described above has all focused on common strains of laboratory rodents. It is important to remember that there are two reasons why an animal may be anxious. It may be predisposed to have an anxious character, for example, through genetic manipulation or selective breeding. This is known as trait anxiety. However, anxiety in response to stress or diet is known as state anxiety, and it is this form of anxiety that is most relevant here. However, it would be interesting to see whether responses to diet differ depending on the initial trait anxiety of the animal and, indeed, whether diet could be anxiolytic under these conditions. The choice of stock diets fed in such studies could then become important in terms of alleviating the side effects of drugs and improving animal welfare and even lifespan. In terms of diet-regulating anxiety, it is the basal levels of anxiety which are perhaps most interesting, but it will also be important, from the point of view of animal welfare, to establish the extent of the dietary effect on behaviour before challenging the animal to further stress or treatments.

Although research in this area is gathering momentum, a key issue in this and other related areas is the difficulty in comparing the outcomes of trials that involve differences in diet, species, strain, sex and life stage, and so forth, coupled to variation in duration, environment, and outcome measure. These issues have been aired in the context of diet-induced obesity in adult life [73, 74] and also apply to dietary impact on anxiety behaviour. Given the endless possibilities of diets that could be investigated under different feeding schedules, it would be pragmatic to focus on core dietary components in order to build up as complete a picture as possible. Even with the high profile already being afforded diets high in fat and sugar, there are still very large gaps in current knowledge. The effects of high-sugar diets during pregnancy, for example, have to date been studied to only a limited degree. Also, the quantities of fat or sugar needing to be consumed before there is a measurable effect, how long lasting is that effect, and how is it affected by trait anxiety and random stressful events remain to be determined. 

The body of evidence reviewed here strongly supports the contention that diet has the potential to induce or ameliorate anxiety-related behaviour, but it remains to be definitively established how these events are regulated and whether there is potential to use these pathways to identify diets which may be beneficial in promoting healthy energy balance and mental health.

## Figures and Tables

**Table 1:** Common tests of anxiety.

Test	Indicator of anxiety	Additional uses/outcomes
Mazes (T maze, Y maze, elevated plus maze, O maze)	Decreased time in open (unwalled) sections	Activity, speed, and cognition
Light dark box	Decreased time in light box	Activity and speed
Open field	Less time in centre regions	Activity and speed
Sucrose preference	Decreased consumption of sucrose	Anhedonia
Locomotor activity	Decreased activity	Activity, speed, and rearing behaviours
Marble burying	Increased compulsive burying behaviour	Compulsion, impulsivity, and neophobia
Novel object	Decreased exploration of unfamiliar object	Activity, speed, memory, impulsivity, and neophobia
